# Engineering Characteristics of Dredged Sediment Solidified by MSWI FA and Cement Under Different Curing Conditions

**DOI:** 10.3390/ma18112622

**Published:** 2025-06-03

**Authors:** Shucheng Zhang, Haoqing Xu, Xinmiao Shi, Wenyang Zhang, Jinyuan Xu

**Affiliations:** 1 Jiangsu Province Engineering Research Center of Geoenvironmental Disaster Prevention and Remediation, School of Architecture and Civil Engineering, Jiangsu University of Science and Technology, Zhenjiang 212100, China; wyzhangluck@163.com; 2 College of Civil and Transportation Engineering, Hohai University, Nanjing 210024, China; sxmhhhh@163.com (X.S.); 231604010130@hhu.edu.cn (J.X.)

**Keywords:** dredged sediment, MSWI FA, landfill cover materials, curing conditions, mechanical properties, waste utilization

## Abstract

Traditional landfill cover materials have low strength and poor dry–wet durability. Municipal solid waste incineration fly ash (MSWI FA) can be used to partially replace cement solidification dredging sediment (DS). This article investigates the possibility of using MSWI FA and ordinary Portland cement (OPC) composite cured DS as a covering material. The mechanical properties, permeability, and wet–dry durability of the cured system were investigated under the conditions of MSWI FA content ranging from 0% to 60% and OPC content ranging from 10% to 15%. The microscopic mechanism was analyzed by scanning electron microscopy and X-ray diffraction. The results showed that when the OPC and MSWI FA contents were 15% and 20%, respectively, the comprehensive performance of the cured specimens was best after 28 days of natural curing. The unconfined compressive strength reached 1993.9 kPa, and the permeability coefficient decreased to below 1 × 10^−7^ cm/s, fully meeting the requirements for landfill coverage. C-S-H gel is the main strength source of the solidified body, while Friedel salt and ettringite enhance the compactness of the matrix. An excessive moisture environment promotes the water absorption of soluble salts produced by MSWI FA hydration, leading to sample expansion and reduced strength. MSWI FA and OPC cured DS exhibit good compression performance in the intermediate cover system of landfills, and can maintain good engineering performance under periodic dry–wet cycles. This dual strategic synergy solves the hazardous disposal problem of MSWI FA and the resource utilization demand of DS, demonstrating enormous application potential.

## 1. Introduction

The intermediate cover layer of a landfill, as a key barrier to pollution spread, directly relates to the effectiveness of environmental risk prevention and control. Traditional clay cover systems, although widely used, have significant drawbacks. Insufficient mechanical strength easily leads to cracking of the cover layer. Cracking and poor stability under dry–wet cycles accelerate material degradation, and a large amount of high-quality clay resources are consumed, exacerbating the unsustainable use of land resources [1]. As landfill capacities become increasingly strained and environmental standards are raised, the development of new cover materials that combine high engineering performance with environmental adaptability has become an urgent need in geotechnical engineering.

In river and lake dredging projects, a large amount of sediment is generated, with global annual dredging producing hundreds of millions of tons of sediment [2]. These sediments are large in volume, have a high water content, and are difficult to dispose of, thus requiring efficient and environmentally friendly disposal strategies. This method not only occupies limited landfill capacity but also consumes scarce high-quality soil resources. Current solidification methods include dewatering solidification [3], microbial-induced solidification [4], and chemical additive solidification [5]. Some researchers [6] systematically evaluated the application of geotextile dewatering in the treatment of Taihu Lake sediment. By comparing five dewatering agents, it was found that the aluminum–microbial complex was the most effective in accelerating dewatering kinetics. Other researchers [2] studied the performance of OPC-cured DS in seawater, and the results showed that when the samples were immersed in seawater 8 h after mixing, the cured body achieved significant compressive strength after 7 days. In view of this situation, scholars around the world have extensively studied the engineering performance of solidified DS under different environmental conditions [7,8]. In order to improve the curing effect, some researchers [9] studied the influence of sodium silicate, polyurethane, and OPC on the unconfined compressive strength of cured DS. Other researchers [10] have studied the effects of construction waste on the mechanical properties, permeability, and microstructure of solidified sediment under dry–wet cycle conditions. The results showed that the strength of solidified sediment increased by 8.5% to 72.1% with the addition of construction waste. Although these methods can achieve the solidification of DS, they require significant investment and are time-consuming. Developing an effective, environmentally friendly, and low-cost method to convert this waste into engineering materials remains a key challenge.

MSWI FA appears as a byproduct of municipal solid waste incineration, and improper handling can cause ecological damage and health risks [11]. Currently, over 70% of FA is directly landfilled globally; however, using it in engineering construction could bring significant economic and environmental benefits [12]. Some researchers have developed two backfilling techniques (HBT and DWT) that repurpose municipal solid waste incineration residues into self-cementing materials for backfilling salt mine cavities, achieving waste valorization, mitigating surface accumulation, and enhancing underground structural stabilization [13].The S/S method has become the primary and widely adopted technology for the safe and environmentally friendly disposal of MWSI FA [5]. After implementing appropriate harmless disposal technologies, MSWI FA can eventually be stored in landfills [14,15]. Existing research indicates that MSWI FA has a certain degree of reactivity and can partially replace cement under specific conditions [16]. Some researchers [17] found that after using fly ash to replace cement, the compressive strength of cement mortar was not affected when the fly ash content was less than 10%, while the compressive strength decreased when the fly ash content was more than 10%. If MSWI FA is used to replace OPC and then solidify DS as the alternative material to become the middle layer soil covering material of the landfill, it can not only realize the resource cycle of “treating waste with waste”, but also reduce the limitations of traditional covering materials and provide dual environmental and economic benefits.

However, there are still some key bottlenecks in the existing research. When the soil covered by the middle layer needs to withstand harsh conditions such as dryness, moisture, and leachate immersion, how much OPC can MSWI FA replace at most to solidify the sediment? Can the physical and chemical properties of the solidified soil meet the requirements of the middle layer of the landfill site? However, current curing technologies mostly focus on optimizing short-term mechanical properties, lacking systematic analysis of permeability, durability, and microscopic mechanisms.

In order to solve the problems given above, this study proposes using a MSWI FA–OPC collaborative cured DS as the core, and designing multi-scene curing experiments to simulate real environments. Four different MSWI FA blending rates and two OPC blending rates were designed and subjected to standard curing, underwater curing, and natural curing, respectively. After solidification, a series of analyses were conducted on the solidified sediment through unconfined compressive strength tests, permeability tests, wet–dry cycle tests, as well as SEM and XRD tests. These analyses will reveal the roles and solidification mechanisms of MSWI FA and OPC in the solidification process of dredged sediment, and select better solutions. This study provides valuable reference for the safety assessment of using MSWI FA solidified sediment as intermediate cover material in landfills.

## 2. Experimental Materials and Methods

### 2.1. Experimental Materials

**Figure 1:**
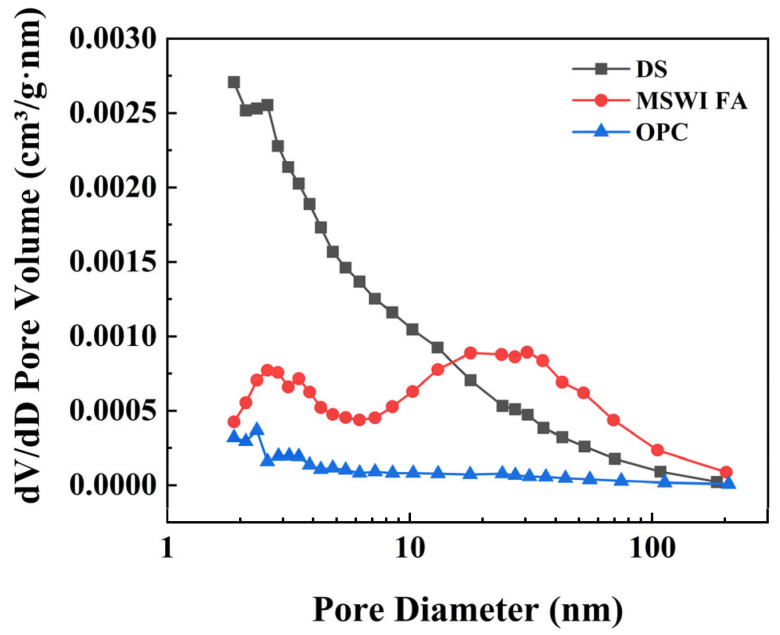
Pore size difference distribution obtained from nitrogen adsorption isotherm curve.

The basic physical parameters of DS and MSWI FA are listed in Table 1. The DS used in this study was collected from a sediment disposal site in Taihu Lake, Wuxi, China, with a specific gravity of 2.09. The liquid limit and plastic limit were approximately 51.2% and 30.3%, respectively, and the natural water content was 129%. It was a typical high-liquid-limit clay. The MSWI FA was sourced from a municipal solid waste incineration power plant in Nanjing, China, exhibiting a specific gravity of 2.54, air-dried wate content of 26.34%, and saturated water content of 52.63%. The OPC used was P.O 42.5 grade cement produced by Yangchun Cement Co., Ltd. in Zhucheng City, China. Figure 1 shows the differences in pore size distribution among three raw materials. Table 2 presents the specific surface areas of DS, MSWI FA, and OPC before and after adsorption.

**Figure 2:**
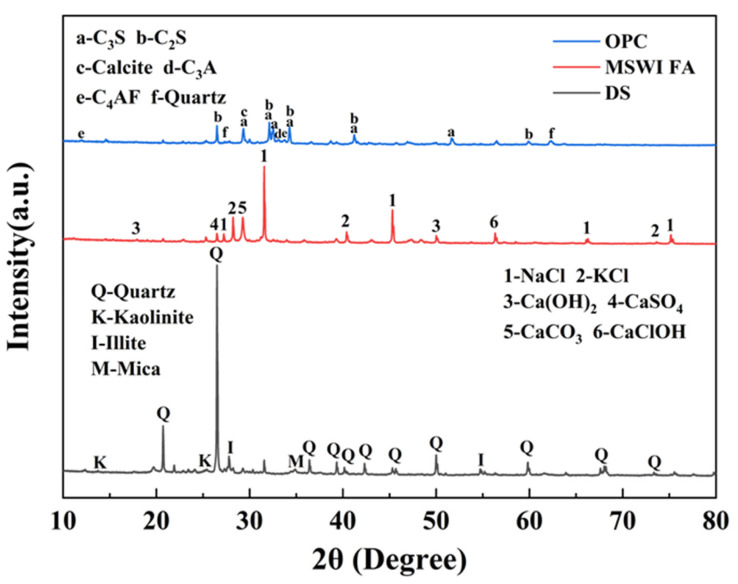
XRD of raw materials.

The XRD patterns of MSWI FA, DS and OPC are shown in Figure 2. The crystalline peaks of MSWI FA were identified as NaCl (PDF#04-007-9715), KCl (PDF#97-002-8938), Ca(OH)_2_ (PDF#97-001-5471), CaSO_4_ (PDF#99-000-0120), CaCO_3_ (PDF#04-007-8659)_,_ CaClOH (PDF#97-006-2041). The main mineral phases of DS were quartz (SiO_2_, PDF#97-003-9830), kaolinite (Al_4_(OH)_8_(Si_4_O_10_), PDF#97-024-5888), mica ((K,H_3_O)Al_2_(Si_3_Al)O_10_(H_2_O,OH)_2_, PDF#99-000-1661) and illite (KAl_2_(Si_3_Al)O_10_(OH)_2_ PDF#00-043-0685), while OPC primarily consisted of C_3_S (PDF#00-055-0740), C_3_A (PDF#00-032-0148), C_4_AF (PDF#97-009-7971), C_2_S (PDF#97-009-4742), Calcite (CaCO_3_ (PDF#04-007-8659)) and quartz (SiO_2_, PDF#97-003-9830).

**Figure 3:**
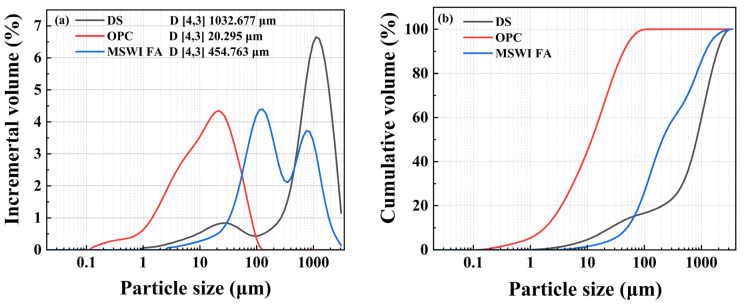
Particle size distribution curves: (**a**) incremental volume, (**b**) cumulative volume.

The particle size distribution of the raw materials was measured using a laser diffraction particle size analyzer (Malvern Mastersizer 3000, Malvern Panalytical, Malvern, UK) as shown in Figure 3. Three samples had distinct peaks at particle sizes of 1032.677 μm, 20.295 μm, and 454.763 μm. The chemical compositions of MSWI FA, DS, and cement were measured by X-ray fluorescence (XRF) spectroscopy (Rigaku ZSX Primus IV, Tokyo, Japan), with results shown in Table 3. Both OPC and MSWI FA were high in CaO (55.03% and 33.98%, respectively). The SiO_2_ content in OPC was 22.14%, while MSWI FA had only 2.84%, and it also contained 24.78% chlorine. The chemical composition of DS was mainly SiO_2_ (63.66%) and Al_2_O_3_ (15.47%).

### 2.2. Sample Preparation

MSWI FA is first dried in an oven at 105 °C for 24 h, then ground with a grinder and passed through a 300-mesh sieve, with the sieved powder sealed and stored for later use. The DS is mechanically stirred to a homogeneous state, with an initial moisture content of 129%. Place the MSWI FA, DS, and OPC in a planetary mixer according to the predetermined mass ratio and mix for 3 min to ensure sufficient contact between the cementitious materials and the matrix. The mixed material is filled into the mold in three batches, with each batch being vibrated to eliminate internal voids. Finally, the surface is leveled, and standard curing (in a standard curing box at 20 ± 2 °C, RH ≥ 95%), underwater curing (fully submerged in a water tank), and natural curing (in a 24 °C indoor environment) are conducted for 28 days to simulate the actual service environment of landfill cover layers.

**Figure 4:**
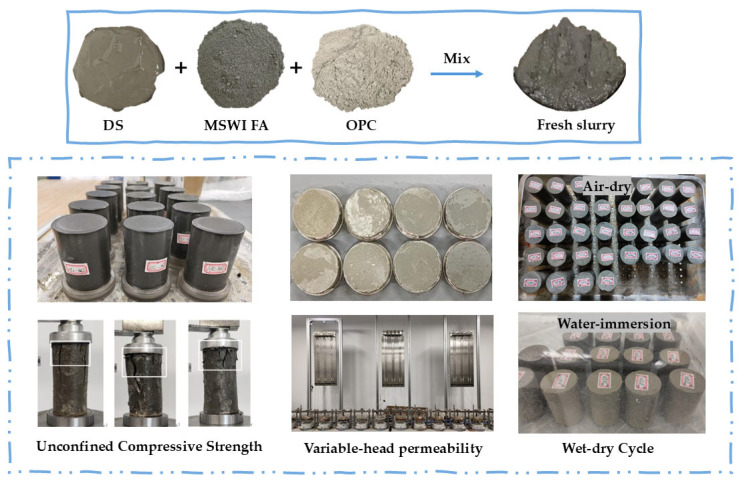
Fresh slurry and various macroscopic test samples.

The proportions of OPC and MSWI FA are calculated as a percentage of the mass of the DS. For example, C10F0 means 10% OPC and 0% MSWI FA. Preliminary tests show that when the OPC content is 5%, the sample becomes loose and lacks load-bearing ca-pacity due to insufficient hydration. When the MSWI FA content is 80% or 100%, the mixture struggles to form a continuous framework because there is no cementing phase, and the strength is almost zero. These findings set key limits for choosing the parameters: 10% OPC provided adhesion to the sample, and 60% MSWI FA gave the sample strength. The 10–15% OPC range best balanced the cost, and the four MSWI FA amounts (0–60%) looked at their function from OPC replacement (0%) to extra binder (20–40%) and main aggregate (60%). The fixed DS mass (500 g) kept the filler content the same. The OPC content was set at 10% and 15%, and the MSWI FA content at 0%, 20%, 40%, and 60%, with a fixed DS mass of 500 g. The specific mix ratios are shown in Table 4. Figure 4 shows the samples of fresh slurry and various macro-scale tests.

### 2.3. Experimental Methods

#### 2.3.1. Unconfined Compressive Strength Tests

The unconfined compressive strength (UCS) test was conducted with reference to the Chinese standard (GB/T50123-2019). An unconfined compression apparatus (model of WKY-1) produced by Nanjing Soil Instrument Factory (Nanjing, China) was used to measure the strength of cylindrical specimens with an inner diameter 3.91 cm of and a height of 8 cm. During loading, a constant displacement rate of 1 mm/min was maintained, and load-deformation data were collected in real-time through built-in force sensors and displacement transducers until structural failure occurred. The displacement sensor is a dc-lvdt sensor produced by Fuxin Fuchuan Sensor Co., Ltd. (Shanghai, China), with an accuracy of 0.2. To ensure reliability, three parallel specimens were prepared for each mix proportion, and the arithmetic mean of the test results was taken.

#### 2.3.2. Variable-Head Permeability Tests

Refer to the Chinese standard (GB/T50123-2019) for conducting variable head permeability tests. Using the 16-channel variable water head permeameter produced by Nanjing Soil Instrument Factory (Nanjing, China), cylindrical samples (with an inner diameter of 6.18 cm and a height of 2 cm) were subjected to standard curing for 2 days to achieve the initial strength before conducting the permeability test. During the test, a pre-consolidation pressure of 12.5 kPa was applied and maintained for 24 h to eliminate the initial void ratio differences. During the 28-day permeability test, the changes in hydraulic head were continuously recorded, and the permeability coefficient was calculated using Formula (1):

(1) $$K=2.3\frac{aL}{At}lg\frac{H_{1}}{H_{2}}$$

where a is the cross-sectional area of the variable head pipe (cm^2^), *L* is the height of the sample (cm), *H*_1_ and *H*_2_ are the initial and final head differences (cm), *A* is the cross-sectional area of the sample (cm^2^), and *t* is the head drop time (s). The average of three measurements is taken for each set of data.

#### 2.3.3. Wet–Dry Cycle Tests

The dry–wet alternating environment of landfill cover layers was simulated as described below. The dry–wet cycle test refers to the test methods proposed by Kamei et al. [18] and the Japan Highway Association [19]. The cured samples were placed in a well-ventilated indoor area at 20 ± 1 °C for 24 h. Afterwards, they were immersed in water for 24 h to complete a single cycle. After wiping off the surface moisture, its mass loss rate was determined, and then UCS tests were conducted to calculate the strength loss rate. Each sample underwent five wet–dry cycles, and the strength loss rate and the mass loss rate were calculated as follows:

(2) $$Strength\;loss\left(\%\right)=\frac{S_{initial}-S_{final}}{S_{initial}}\times 100\%$$

(3) $$Mass\;loss\left(\%\right)=\frac{M_{initial}-M_{final}}{M_{initial}}\times 100\%$$

In the formula, *S_initial_* and *S_final_* are the UCS of the sample before the wet–dry cycle and the UCS of the sample after the nth cycle (kPa), *M_initial_* and *M_final_* are the mass of the sample before the wet–dry cycle and the mass of the sample after the nth cycle (g).

#### 2.3.4. X-Ray Diffraction (XRD)

After the unconfined compressive strength test, the crushed sample was placed into a 105 °C oven to dry for 24 h, then ground into powder using a mortar and pestle and sieved through a 300-mesh screen. The Japanese Rigaku SmartLab SE (Rigaku, Tokyo, Japan) diffractometer was used to analyze the phase composition (refer to GB/T 30904-2014 standard); the scanning range is 10–80° (2θ), with a step size of 0.02° and a scanning speed of 5°/min.

#### 2.3.5. Scanning Electron Microscopy (SEM)

After the unconfined compressive strength test, the crushed sample was placed in a 105 °C oven to dry for 24 h; then, a piece of the crushed sample with a length, width, and thickness all less than 1 cm was taken as the test sample. Due to the poor conductivity of the sample, gold was sputtered onto its surface. The German ZEISS Sigma 360 scanning electron microscope (Carl Zeiss AG, Oberkochen, Germany) was used to observe the microstructure (refer to JYT0584-2020 standard).

## 3. Results

### 3.1. Mechanical Properties

By comparing the stress–strain evolution patterns of cured samples under standard curing, underwater curing, and natural curing conditions, we can better understand the mechanical performance stability of OPC-MSWI FA co-cured DS under different working conditions. The peak stress corresponding to the stress–strain curve is the UCS, which reflects the load-bearing capacity of the material under compression. The failure characteristics of the specimen show that the initial vertical crack at the top gradually expands and evolves under the guidance of tensile stress, finally forming a tensile fracture.

**Figure 5:**
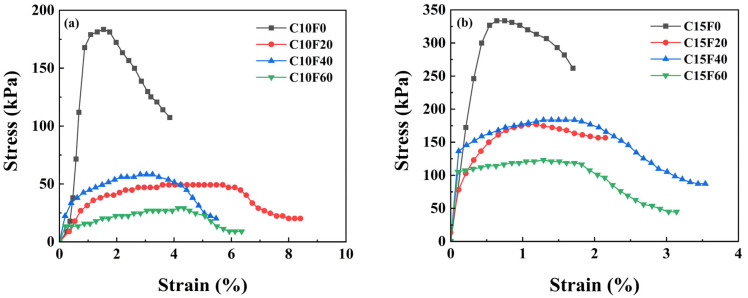
Stress–strain curves of solidified sediment under standard curing conditions: (**a**) 10% OPC, (**b**) 15% OPC.

Figure 5 shows the stress–strain curve of the sample after standard curing for 28 days. Under the condition of 10% OPC content, the UCS of the sample without MSWI FA reached 184.7 kPa, exhibiting brittle failure. As the MSWI FA content increases, the material strength gradually decreases. For example, when the MSWI FA content reaches 20%, the UCS drops to 49.7 kPa (a decrease of 73.1%), exhibiting plastic failure. Moreover, when the OPC content is increased to 15%, the UCS of the sample with the same 20% MSWI FA content recovers to 183.5 kPa (an increase of 72.9%), revealing the crucial role of cement content in strength recovery. However, when the MSIW FA content increases to 60%, the sample strength is only 27.0 kPa, which is below the landfill requirement of 50 kPa strength threshold. It can be seen that while the incorporation of MSIW FA under standard curing conditions can improve the deformation performance of the material, it will reduce the strength of the solidified body. Therefore, it is necessary to control the proportion of cementitious materials to achieve a balance between mechanical performance and engineering requirements.

**Figure 6:**
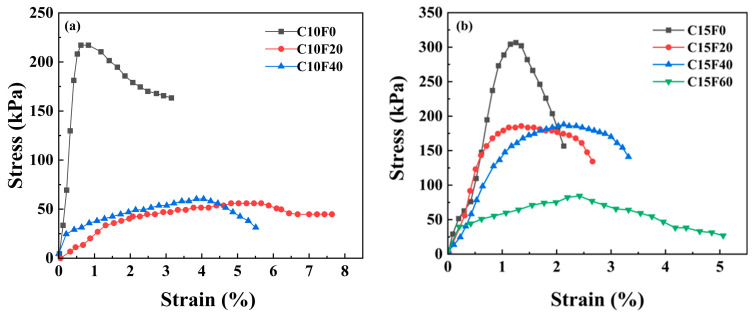
Stress–strain curves of the solidified sediment under submerged curing conditions: (**a**) 10% OPC, (**b**) 15% OPC.

Figure 6 shows the stress–strain curve of the specimen after 28 days of underwater curing. When the OPC content is 10% and the MSWI FA content is 60%, the sample exhibits abnormal softening after curing (moisture content > 90%), and its UCS is below the detection limit, resulting in the absence of a stress–strain curve in Figure 5a. When the OPC content is 15%, as the MSWI FA content increases from 0% to 60%, the unconfined compressive strength peak of the solidified body significantly decreases from 306.6 kPa to 84.4 kPa (a reduction of 72.5%). The strength degradation is mainly attributed to the continuous leaching of soluble Na^+^, K^+^, and other alkali metal ions in the MSWI FA during the hydration process, leading to the formation of pores within the solidified body.

**Figure 7:**
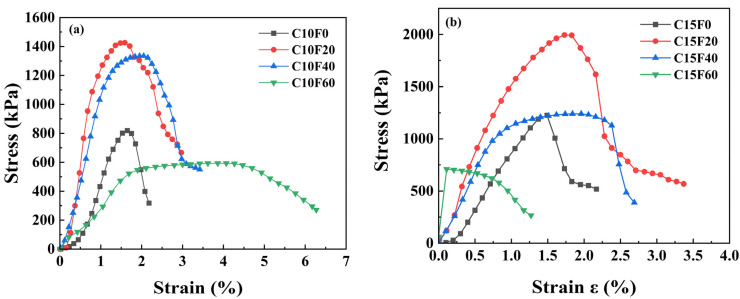
Stress–strain relationships of the solidified sediment under natural curing conditions: (**a**) 10% OPC, (**b**) 15% OPC.

Figure 7 shows the stress–strain curve of the sample after natural curing for 28 days. Taking the group with 10% OPC content as an example, during the process of increasing the MSWI FA content from 0% to 20%, the UCS increased from 819.0 kPa to 1424.9 kPa, an increase of 74%. However, with further increases in MSWI FA content, the UCS decreased. When the MSWI FA content reached 60%, the compressive strength significantly decreased, indicating that excessive MSWI FA introduction inhibits the formation of C-S-H gel. It can be seen that the unsaturated state formed by natural curing can effectively promote the growth of cementitious products, significantly enhancing the compressive strength of the solidified body.

### 3.2. Permeability

**Figure 8:**
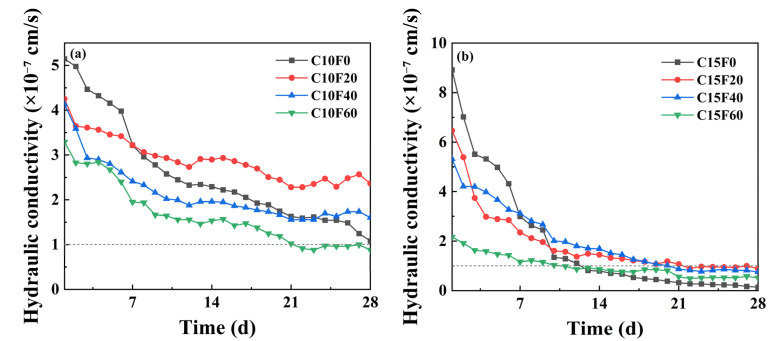
Variation in permeability coefficient with curing age: (**a**) 10% OPC, (**b**) 15% OPC.

As a key barrier layer in the landfill capping system, the hydraulic conductivity of the cover soil directly determines its efficiency in impeding rainwater infiltration and leachate migration. Figure 8 shows the evolution of the permeability coefficient of different mix ratio samples over a 28-day curing period. The permeability coefficients of all mix samples experienced a rapid decrease during the first 14 days of curing, and then gradually stabilized. This is because, in the early stage (before 14 days), the samples, although having some strength, had not yet fully completed the OPC hydration reaction, and the MSWI FA had strong water absorption properties. However, after 14 days, the hydration product network had formed a continuous framework, and the material structure tended to stabilize. After 28 days of permeability testing, the permeability coefficients of C10F0, C10F20, and C10F40 were all greater than 1 × 10^−7^ cm/s. However, when the OPC content is increased to 15%, the permeability coefficients of the samples all meet the standards, indicating that the sample with 15% OPC content has the best density. According to the previous text, the UCS value of the C15F20 sample is the highest, corresponding to k = 0.88 × 10^−7^ cm·s^−1^, which meets the US EPA’s permeability threshold for landfill cover materials (k < 1 × 10^−7^ cm/s) [20].

### 3.3. Wet–Dry Durability

**Figure 9:**
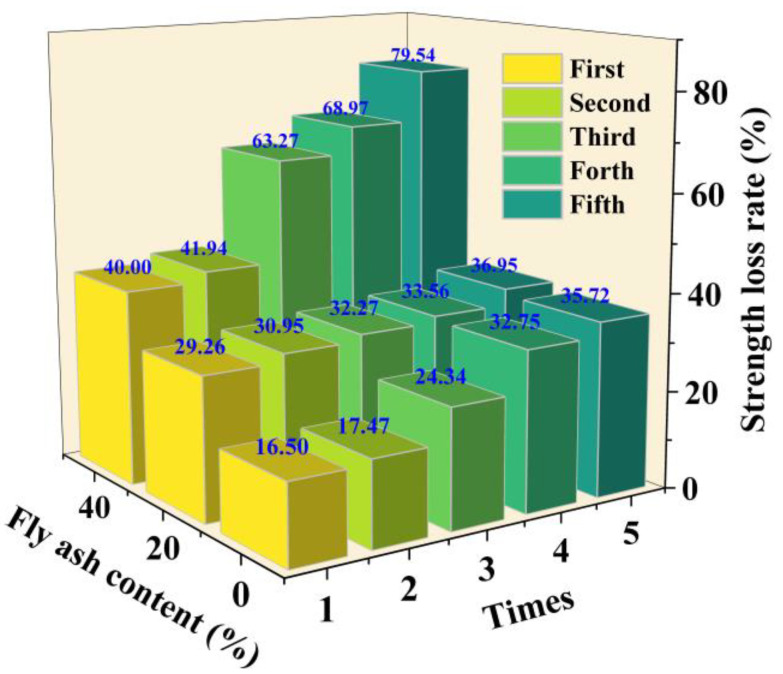
Strength loss rate of specimens under different wet–dry cycling counts.

Due to changes in natural environmental conditions such as rainfall, evaporation, and groundwater level fluctuations, the mechanical stability of solidified dredged sediment in alternating dry and wet environments directly determines its service life in engineering applications. Dry–wet cycle tests were conducted on samples cured for 28 days under standard conditions, with the OPC content at 10% and the MSWI FA content at 0%, 20%, and 40%. Three cases were tested, and the samples with 60% MSWI FA content were completely discarded after the cycle due to total loss of strength. The UCS loss rate before and after the wet–dry cycles is shown in Figure 9. The strength loss rate of each mix ratio sample after a single dry–wet cycle exceeds 15%, and the strength loss rate increases with the number of dry–wet cycles. Under the same number of dry–wet cycles, as the amount of MSWI FA increases, the strength loss rate of the samples gradually increases. For example, when the MSWI FA content is 40%, the strength loss rate sharply increases to 79.54% after five cycles, which is 1.15 times higher than that of the sample with 20% MSWI FA content (29.26–36.95%).

**Figure 10:**
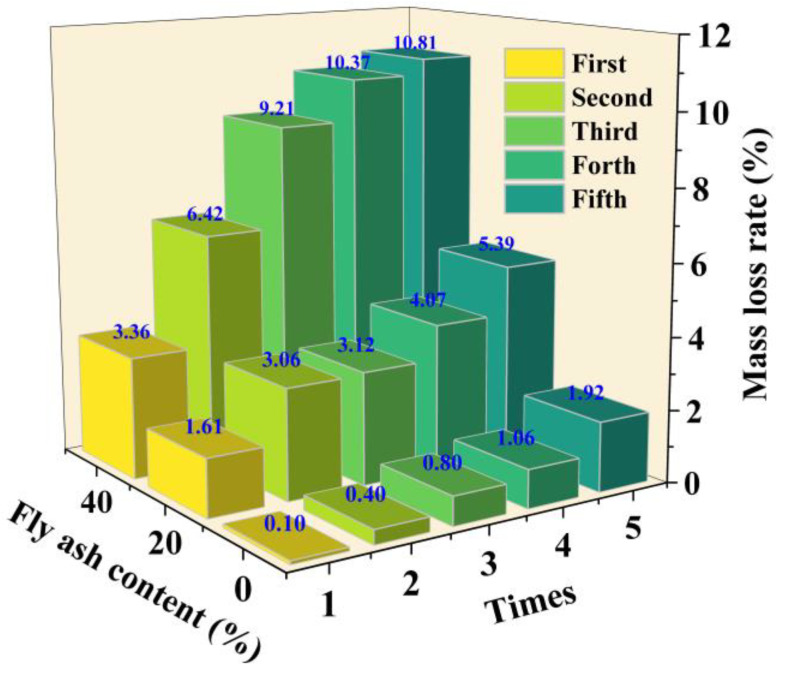
Mass loss rate of specimens under different wet–dry cycling counts.

**Figure 11:**
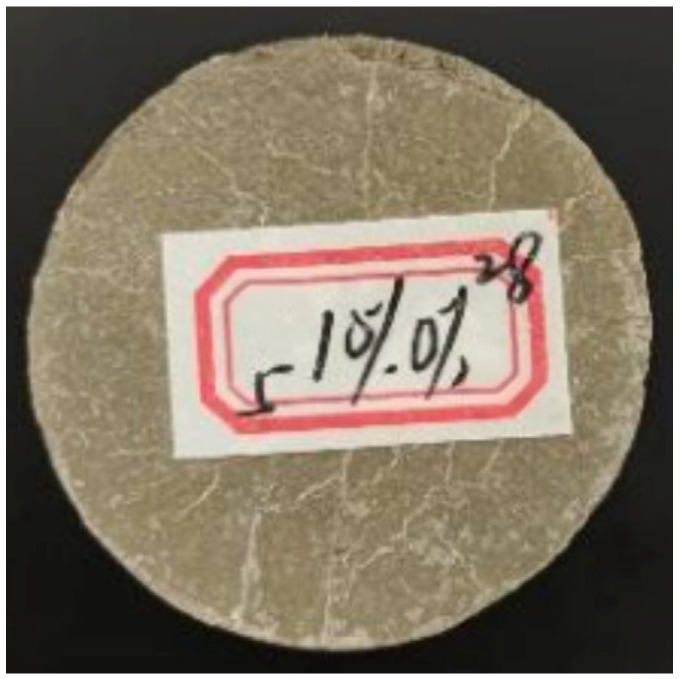
Image of the specimen after wet–dry cycle.

Figure 10 shows the mass loss rate of the samples under different dry–wet cycle counts. The quality loss rate increases with the increase in dry–wet cycles, which is consistent with the trend of strength loss rate, indicating that the two may have a mutual influence. The quality loss rate of samples with 0% MSWI FA content is less than 2%. This indicates that the wet–dry cycle has little effect on pure cement-based materials. In contrast, the quality loss rates of specimens with MSWI FA content of 20% and 40% were 5.39% and 10.81%, respectively. It is speculated that the wet–dry cycle promotes the repeated dewatering of solidified sediment, leading to a continuous increase in porosity and a decrease in matrix suction. After multiple cycles, the sediment shrinks to a critical volume, and its water absorption and expansion are limited by the skeleton, resulting in internal cracks and weakened particle bonding to form a loose structure, leading to a decrease in strength. In addition, cracks were observed in the sample after wet–dry cycling (as shown in Figure 11). This phenomenon may be caused by the continuous leaching effect of soluble salts in MSWI FA. During the adhesion process, NaCl crystals repeatedly dissolve and recrystallize at the crack site. This process leads to the dissolution of crack propagation material, ultimately resulting in quality loss.

### 3.4. Crystalline Phases

**Figure 12:**
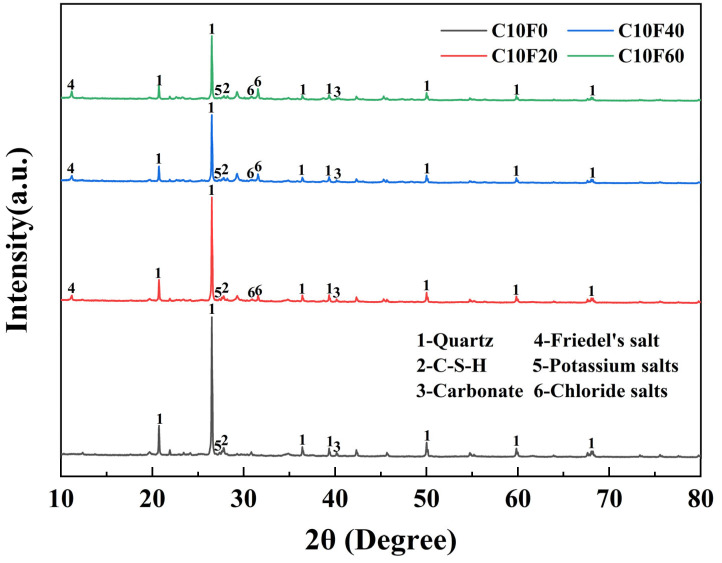
XRD pattern.

Figure 12 shows the XRD analysis results of samples with different MSWI FA contents after standard curing when the cement content is 10%. From the figure, it can be seen that the main phases are quartz (PDF#97-003-9830), C-S-H (PDF#97-003-1250), carbonate (PDF#97-002-0179), Friedel’s salt (PDF#01-078-1219), potassium salt (PDF#97-001-8014), and chloride salt (PDF#01-070-2509) [20,21]. Among them, the diffraction peak of quartz phase at 26.6° (2θ) is the most obvious. At 40.54° (2θ), a diffraction peak of carbonate was detected [22], which is due to the carbonation reaction of MSWI FA with air during the curing process, forming calcium carbonate. Furthermore, the diffraction peak intensity of C-S-H near 27.4° (2θ) decreased with the increase in MSWI FA content, a trend consistent with the variation pattern of unconfined compressive strength under standard curing conditions. This indicates that the incorporation of MSWI FA inhibits the formation of crystalline C-S-H phases, thereby leading to a decline in mechanical performance. Notably, the characteristic peak of Friedel’s salt appearing at 11.4° (2θ) confirms the presence of a chloride immobilization mechanism within the curing system [23]. The main components of MSWI FA are CaO and Cl, it will react with the Ca(OH)_2_ generated by cement hydration as follows: Al_2_O_3_ + Ca(OH)_2_ + CaCl_2_ + H_2_O → 3CaO·Al_2_O_3_·CaCl_2_·10H_2_O. As the content of MSWI FA in the sample increases, the soluble salts also increase accordingly. Taking potassium salt (2θ = 27.3°) and chloride salt (2θ = 31.56°) as examples, their peak intensity ratios (I/I_0_) increased from 0.12 in C10F0 to 0.58 in C10F60.

### 3.5. Micromorphology

**Figure 13:**
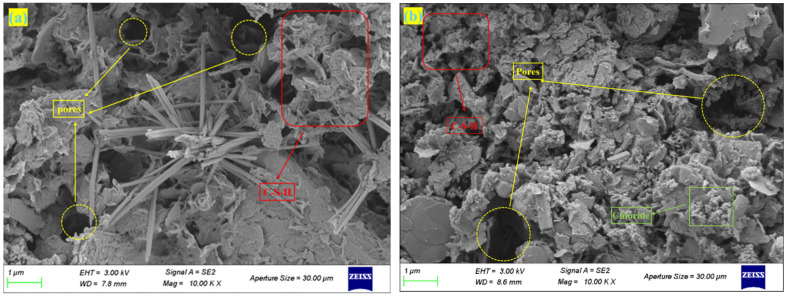

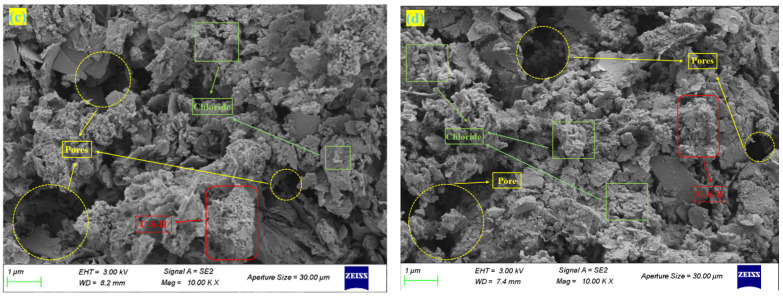
SEM images of the cured specimens: (**a**) C10F0, (**b**) C10F20, (**c**) C10F40, (**d**) C10F60.

Figure 13 shows the SEM images of sediment stabilized with 10% OPC and varying MSWI FA contents after 28 days of standard curing. C10F0 displays a dense structure dominated by typical cement hydration products. The flaky and granular sediment matrix is fully encapsulated by a mesh-like cementitious material [24], with significantly lower porosity compared to other groups. The microstructural compactness is positively correlated with its UCS. C10F20 and C10F40 exhibit significant structural deterioration, characterized by reduced hydration product formation and increased porosity relative to C10F0. Chloride salts are observed to inhibit the hydration processes of C_3_S and C_2_S, thereby hindering the formation of C-S-H gel. Their mechanical properties mainly stem from the physical filling effects of hexagonal Ca(OH)_2_ plates and Friedel’s salt [25]. C10F60 reveals distinct phase separation in the microstructure, with free chloride salts accumulating in pores to form micron-sized crystalline aggregates. Structural loosening leads to a sharp decrease in UCS.

## 4. Discussion

The mixing of OPC, MSWI FA, and DS initiates the hydrolysis reaction of C_3_S and C_2_S in OPC and CaO in MSWI FA, which rapidly releases Ca^2+^ and OH⁻ ions (Formula (4)) [26], generating a large amount of Ca(OH)_2_ supersaturated precipitated crystals (Formula (5)). At the same time, Na^+^ and K^+^ also hydrolyze to create an alkaline environment that promotes the depolymerization of glassy SiO_2_ and Al_2_O_3_ in MSWI FA [27]. The dissociated silicoaluminate reacts with Ca^2+^ through a polycondensation reaction to form C-S-H and C-A-H gels [28]. The main cementitious phase forms a network structure and a dense structure by encapsulating particles, thereby enhancing the strength of the solidified body (Formulas (6) and (7)). In the system, SO_3_^2−^ reacts with Ca(OH)_2_ to form expansive ettringite (AFt) (Formula (8)), Chloride salt exists in free state in the sample, while Cl^−^ replaces OH⁻ in C-S-H to form Friedel’s salt, both of which synergistically optimize the microstructure (Formula (9)) [29]. However, Friedel salts are a process product that disappears with increasing curing time. Excess free chloride salts compete with OH⁻ for adsorption on the surface of cement particles, hindering water molecules from contacting active sites [30]. Moreover, excessive Cl^−^ preferentially reacts to form Friedel salts, consuming most of the aluminum phase, resulting in a decrease in the production of ettringite (AFt). The absence of AFt as a key component of the early strength skeleton directly weakens the formation efficiency of the cementitious network. The remaining chlorides will be encapsulated by the formed C-S-H or precipitate freely, forming a white crystalline mixture with potassium salts and sulfates in the sample. Under non-sealed curing conditions, Ca(OH)_2_ carbonizes to form CaCO_3_ crystals (Formula (10)). Its deposition, along with the gel products, fills the pores, forming a multi-scale enhancement effect [31]. 

In the natural curing system during the drying phase of the dry–wet cycle, the inability to continuously supply reaction water hinders the hydration of OPC and the hydrolysis of MSWI FA, resulting in a reduced formation of Ca(OH)_2_ compared to standard curing and underwater curing, and a lower degree of SO_3_^2−^ reaction leading to insufficient formation of AFt. These moisture constraint conditions also inhibit the migration and crystallization of soluble salts such as Cl^−^ and K⁺, this leads to a significant weakening of the salt crystallization damage effect [32]. The carbonation reaction during the process exhibits a two-stage characteristic: initially, residual Ca(OH)_2_ is consumed to form CaCO_3_, and later, C-S-H gel is eroded and decalcified to form amorphous SiO_2_ (Formula (11)) [33]. As the reaction progressed, the decrease in specimen moisture content caused reduced interparticle spacing and enhanced van der Waals forces. This physical strengthening effect enhanced the compressive strength, but also caused the resulting shrinkage cracks to become the primary cause of strength and mass reduction during the wetting phase [8]. Based on previous studies, the leaching amount of Zn and Cu in sediments and MSWI fly ash is greater than that of other heavy metals, and the mobility of Zn and Cu is greater than 12.5, indicating that the mobility of Zn and Cu is low and safe [34]. The infiltration of external moisture leads to the cyclic dissolution of Friedel salts and other soluble salts, which then migrate to the surface or pores (Formula (12)) [35]. Moreover, during underwater immersion, decalcification of the C-S-H gel (Ca^2+^ leaching) occurred, transforming it into non-cementitious substances with low calcium-to-silicon ratios, and thereby reducing cementing capacity.

C_3_S/C_2_S/C_3_A + H_2_O → CSH + Ca(OH)_2_ (4)

CaO + H_2_O → Ca(OH)_2_ (5)

Ca(OH)_2_ + SiO_2_ + H_2_O → C-S-H (6)

Ca(OH)_2_ + Al_2_O_3_ + H_2_O → C-A-H (7)

Ca(OH)_2_ + SiO_2_ + H_2_O + SO_3_ + Al_2_O_3_ → CaO•Al_2_O_3_•CaSO_4_•H_2_O (AFt) (8)

Ca(OH)_2_ + CaCl_2_ + Al_2_O_3_ + H_2_O→CaO•Al_2_O_3_•CaCl_2_•H_2_O (Friedel’s salt) (9)

Ca(OH)_2_ + CO_2_ → CaCO_3_ + H_2_O (10)

C-S-H + CO_2_ → CaCO_3_ + SiO_2_ + H_2_O (11)

Friedel’s salt + H_2_O → CaCl_2_ + AlCl_3_ + H_2_O (12)

## 5. Conclusions

This paper systematically investigated the effects of MSWI FA and OPC dosage, along with three curing conditions, on the strength and permeability of solidified DS, yielding the following key conclusions:

(1) Curing conditions and material ratios jointly govern mechanical performance. Under natural curing, appropriate FA content promoted cementitious reactions, with the C15F20 specimen achieving a peak UCS of 1993.9 kPa (62.6% higher than the pure cement group). However, excessive FA (≥40%) caused abrupt strength reduction. Under standard or underwater curing, FA incorporation reduced strength but improved deformation performance, though increasing the OPC dosage from 10% to 15% restored strength. This indicates that FA’s effect on strength is condition-dependent, and its optimal dosage must be determined holistically based on curing environment requirements.
(2) The OPC content is a key factor in controlling the permeability coefficient. When the OPC content was 10%, regardless of whether the MSWI FA content is 0%, 20%, or 40%, the permeability coefficient of the samples is higher than 10^−7^ cm/s, which does not meet the requirements for landfill cover materials; however, when the OPC content is increased to 15%, the permeability coefficients under all FA contents dropped below 10^−7^ cm/s, with the permeability coefficient of C15F20 being 0.88 × 10^−7^ cm/s, meeting the U.S. EPA standards.
(3) Under wet–dry cycling, the strength and mass loss rate of solidified sediment increased significantly with higher FA content. At 10% OPC dosage, specimens with 0%, 20%, and 40% FA exhibited strength loss rates exceeding 35.72%, 36.95%, and 79.54% after five cycles, respectively, and mass loss rates reaching 1.92%, 5.39%, and 10.81%. Results indicate that FA content critically affected material durability under wet–dry cycles, and high dosages (≥20%) significantly reduced engineering service life.
(4) As MSWI FA content increased, C-S-H gel formation was inhibited by chloride salts, resulting in higher porosity and looser structure of the solidified body. At 60% MSWI FA dosage, free chloride salts accumulated in pores, forming micron-scale aggregates and triggering phase separation, leading to severe microstructural degradation. XRD and SEM results confirmed that soluble salts (e. g., K^+^, Cl^−^) introduced by FA not only inhibited cementitious reactions but also exacerbated pore expansion through crystallization–dissolution cycles.

The partial replacement of cement with MSWI FA to solidify dredged sediment as landfill cover material is possible. However, its practical use needs to consider environmental challenges. Leachate from landfills can cause erosion, which may affect the long-term stability of the materials. More research is needed in the future.

## Figures and Tables

**Table 1:** Physical parameters of DS and MSWI FA.

DS		MSWI FA	
Specific gravity	2.09	Specific gravity	2.54
Liquid limit (%)	51.2	Air-dry water content (%)	26.34
Plastic limit (%)	30.3	Saturated water content (%)	52.63
Plasticity index	20.9	Bulk density (g/cm^3^)	1.01
Natural water content (%)	129	Particle size (mm)	0–5

**Table 2:** Specific surface area before and after adsorption of DS, MSWI FA, and OPC.

Thermophysical Propertiese	DS	MSWI FA	OPC
BET Surface Area (m^2^/g)	20.6539	18.0049	1.9086
Micropore volume (cm^3^/g)	0.001241	0.002790	0.000046

**Table 3:** Chemical composition of experimental materials (% mass).

Materials	SiO_2_	Al_2_O_3_	Fe_2_O_3_	Na_2_O	K_2_O	MgO	CaO	SO_3_	Cl	LOI
DS	63.66	15.47	5.20	2.03	2.68	1.83	4.77	1.11	1.71	1.54
MSWI FA	2.84	0.87	0.65	17.76	7.55	1.66	35.93	6.14	24.78	1.82
OPC	23.07	6.66	5.31	0.37	1.01	3.33	55.03	3.84	0.12	1.26

**Table 4:** Mixture proportion.

Number	OPC (g)	FA (g)	DS (g)	Ratio (PC:FA:DS)
C10F0	50	0	500	1:0:10
C10F20	50	100	500	1:2:10
C10F40	50	200	500	1:4:10
C10F60	50	300	500	1:6:10
C15F0	75	0	500	3:0:20
C15F20	75	100	500	3:4:20
C15F40	75	200	500	3:8:20
C15F60	75	300	500	3:12:20

## Data Availability

The original contributions presented in this study are included in the article. Further inquiries can be directed at the corresponding authors.
